# Cardiovascular complications of COVID-19

**DOI:** 10.1172/jci.insight.148980

**Published:** 2021-07-08

**Authors:** Farnaz Farshidfar, Navid Koleini, Hossein Ardehali

**Affiliations:** Feinberg Cardiovascular Research Institute, Feinberg School of Medicine, Northwestern University, Chicago, Illinois, USA.

## Abstract

The emergence of the novel SARS coronavirus 2 (SARS-CoV-2), the causative agent of coronavirus disease 2019 (COVID-19), has resulted in an unprecedented pandemic that has been accompanied by a global health crisis. Although the lungs are the main organs involved in COVID-19, systemic disease with a wide range of clinical manifestations also develops in patients infected with SARS-CoV-2. One of the major systems affected by this virus is the cardiovascular system. The presence of preexisting cardiovascular disease increases mortality in patients with COVID-19, and cardiovascular injuries, including myocarditis, cardiac rhythm abnormalities, endothelial cell injury, thrombotic events, and myocardial interstitial fibrosis, are observed in some patients with COVID-19. The underlying pathophysiology of COVID-19–associated cardiovascular complications is not fully understood, although direct viral infection of myocardium and cytokine storm have been suggested as possible mechanisms of myocarditis. In this Review, we summarize available data on SARS-CoV-2–related cardiac damage and discuss potential mechanisms of cardiovascular implications of this rapidly spreading virus.

## Introduction

Coronavirus disease 2019 (COVID-19) has resulted in a global pandemic that emerged in 2019 and is the result of infection with the novel enveloped RNA beta coronavirus SARS coronavirus 2 (SARS-CoV-2). The first cases of the disease were identified in Wuhan, China, in late 2019, and the disease rapidly spread throughout the world, infecting more than 168 million individuals and causing 3.5 million deaths worldwide as of May 28, 2021 (1).

SARS-CoV-2 primarily infects the respiratory system, manifesting a range of clinical presentations, from asymptomatic subclinical infection to severe acute respiratory distress syndrome (ARDS) that requires mechanical ventilation and admission to the intensive care unit (ICU). Although respiratory failure is the primary cause of death, cardiovascular complications, such as acute myocardial injury and myocarditis (2–4), cardiac fibrosis (5), arrhythmias (6), endothelial dysfunction (7), dysautonomia (8), and thrombotic events (9), may also contribute to overall morbidity and mortality of COVID-19 patients. The pathophysiology of the cardiac manifestations in COVID-19 remains to be fully elucidated. Lack of sufficient histological evidence to thoroughly assess cardiac pathologies, especially in cases such as myocarditis in which histological examination is a part of diagnostic criteria, renders our understanding of cardiac manifestations of SARS-CoV-2 limited. Furthermore, medications currently used to treat COVID-19 may also affect the cardiovascular system. The occurrence of cardiovascular manifestations may also influence the severity of COVID-19, and underlying cardiovascular conditions may increase mortality. Thus, understanding the mechanisms of COVID-19–mediated cardiovascular disease may lead to improvement in the treatment and management of these patients. In this Review, we summarize recent studies on COVID-19–related cardiovascular injury and the pathophysiology of cardiovascular manifestations.

## SARS-CoV-2 entry into host cells

**Figure 1:**
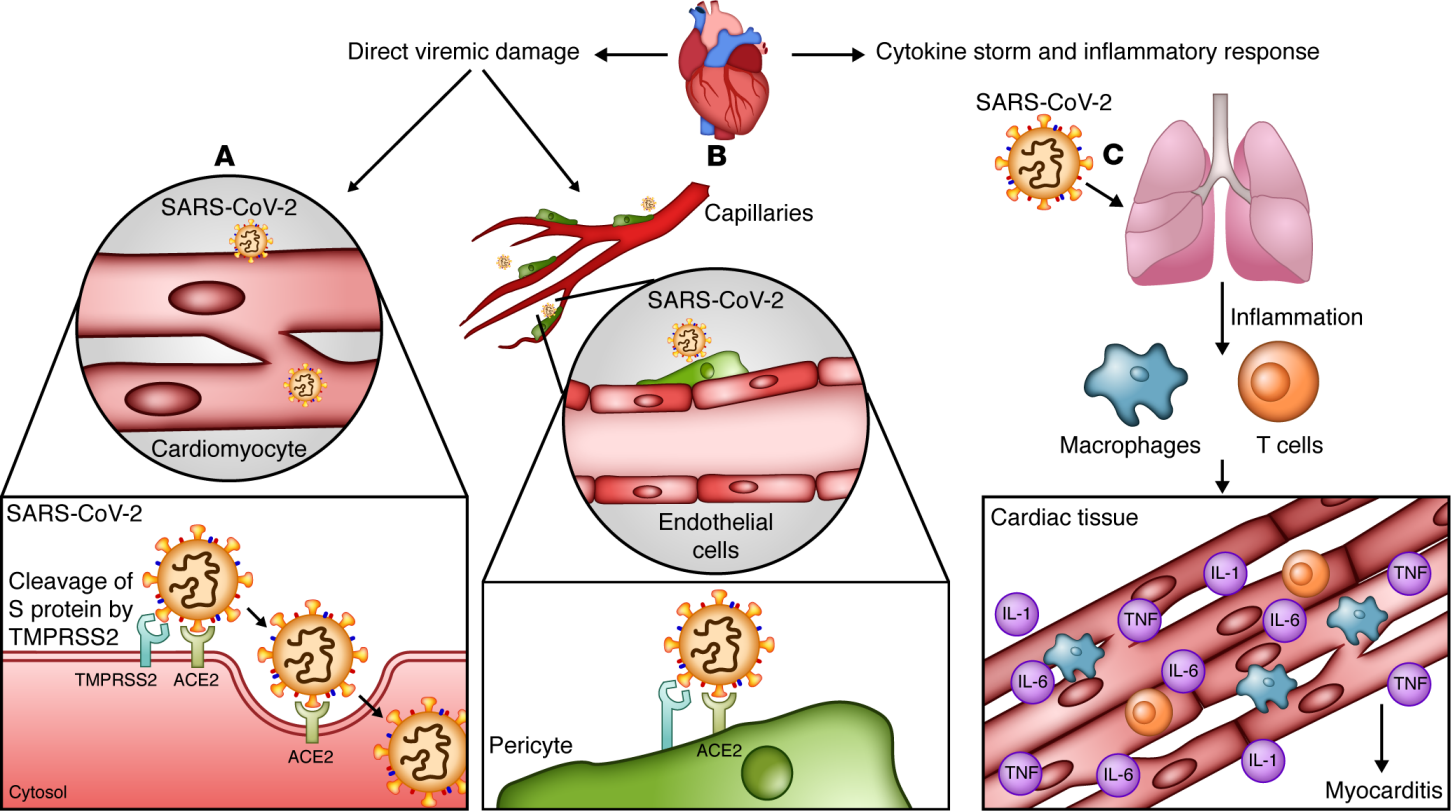
Possible mechanisms of acute myocardial injury and myocarditis in SARS-CoV-2 infection. Two main mechanisms by which SARS-CoV-2 causes myocardial injury are direct virus-induced damage and secondary damage due to cytokine storm and inflammatory responses. (**A**) There is evidence that cardiomyocytes express the receptors required for SARS-CoV-2 entry to the cell. Transmembrane protease TMPRSS2 cleaves the SARS-CoV-2 spike (S) protein, thus facilitating its activation and binding to cell-entry receptor angiotensin converting enzyme 2 (ACE2). (**B**) In addition, pericytes, which are abundant in cardiac tissue and required for endothelial cell (EC) function and maintenance, express ACE2. Injury of these cells by SARS-CoV-2 results in EC dysfunction. (**C**) Furthermore, cytokine storm and systemic inflammatory responses initiated by the virus can also lead to cardiac tissue damage and myocarditis. Illustrated by Rachel Davidowitz.

Some of our understanding of receptor recognition by SARS-CoV-2 originated from coronaviruses responsible for prior epidemics, including SARS and Middle East respiratory syndrome (MERS) (10, 11). Structural studies have shown that the transmembrane spike protein (S protein) on the viral surface mediates the entry of SARS-CoV-2 into cells. The receptor-binding domain on the S protein recognizes the host cell receptor, angiotensin converting enzyme 2 (ACE2), which acts as a functional receptor for viral entry (12, 13). Immunolocalization of ACE2 in different human tissues revealed that ACE2 is widely distributed in all organs and is especially abundant in alveolar epithelial cells and enterocytes of the small intestine; i.e., two organs that are in direct contact with the virus as it enters the body (14). Endothelial cells (ECs), cardiomyocytes, and pericytes in the heart also express ACE2, and thus might be direct targets of SARS-CoV-2. S protein binding to ACE2 requires TMPRSS2-mediated proteolysis (15); therefore, expression of both ACE2 and TMPRSS2 is thought to be required for SARS-CoV-2 entry (Figure 1A). Multiple bioinformatic and experimental approaches have been employed to identify cells that coexpress these entry factors. Comprehensive single-cell RNA-Seq (scRNA-Seq) with subsequent evaluation of protein levels and cellular localization using immunohistochemistry revealed high expression of both of these receptors in the lung, particularly in alveolar epithelial type II cells, confirming the lung as the principal target of SARS-CoV-2. Remarkably, cardiomyocytes display the second highest coexpression of ACE2 and TMPRSS2 (19% of cells) (16). In contrast, another study that evaluated multiple scRNA-Seq data sets noted a lack of coexpression of these receptors in cardiomyocytes (17). Whether expression of ACE2 and TMPRSS2 varies between different age groups, sexes, and races needs further investigation.

It has been shown that SARS-CoV-2 viral particles are present within ECs of capillaries. Additionally, in postmortem tissue analysis of COVID-19 patients, diffuse infiltration of mononuclear cells associated with endothelium and apoptosis of ECs were detected, and endothelitis and endothelial dysfunction in cardiac tissue were reported as consequences of SARS-CoV-2 infection (18). These findings suggest that ECs may also be the direct target of the virus via ACE2 (14, 18). ACE2 expression is also highly enriched in cardiac pericytes. Crosstalk between pericytes and ECs plays a major role in EC function and maintenance; therefore, pericyte injury can result in capillary EC dysfunction (Figure 1B). Patients with underlying cardiovascular disease have a high level of ACE2 expression in pericytes and more severe disease (19). Thus, a cardiovascular disease–related increase in ACE2 may explain increased SARS-CoV-2–associated cardiac damage in individuals with baseline cardiovascular disease.

## Cardiovascular complications of COVID-19

### Myocarditis

Myocarditis is an inflammatory disease of the myocardium that presents with a wide range of symptoms (20). Established histological, immunological, and immunohistochemical criteria (called the Dallas criteria) are currently used to diagnose this disease (21). Based on the Dallas criteria, acute myocarditis is defined as “an inflammatory infiltrate of the myocardium with necrosis and/or degeneration of adjacent myocytes not typical of the ischemic damage associated with coronary artery disease” (22). Different infectious and noninfectious triggers can cause myocarditis, although viral infections by coxsackievirus B, adenovirus, parvovirus B19, hepatitis C virus, Epstein-Barr virus, cytomegalovirus, and human herpesvirus 6 are the most commonly identified causes (20). Additionally, postmortem heart biopsies have shown the presence of myocarditis in some HIV-infected patients (23).

SARS-CoV-2–related coronavirus family members, SARS-CoV and MERS-CoV, have also been reported to cause myocarditis (24–26). Coronavirus-related myocarditis was first reported in 1980 in a 43-year-old man with an upper respiratory tract infection who was hospitalized in Helsinki because of prolonged fever, tiredness, and chest pain. The patient was diagnosed with myocarditis, and later, a significant increase in coronavirus-specific antibody was noted in his blood tests, indicating that in addition to initial upper respiratory infection, coronaviruses can cause subsequent myocarditis (27).

Given that early reports of SARS-CoV-2 infection did not histologically assess myocarditis, the prevalence of this complication in COVID-19 patients is not clear. Several studies have demonstrated elevations in cardiac enzymes and alterations in ECG and echocardiography suggestive of acute myocardial injury in COVID-19 patients (6, 28). However, only a small number of these studies provided endomyocardial biopsy (or in some cases autopsy) results to distinguish between sterile myocardial damage and myocarditis.

The first case of a patient with fulminant COVID-19–related myocarditis was reported in a 63-year-old male with no history of heart disease or hypertension who initially presented with symptoms consistent with pneumonia. Further blood tests revealed high IL-6 and elevated levels of myocardial injury markers, including troponin I, myoglobin, and N-terminal brain natriuretic peptide (NT-BNP). On echocardiography, an enlarged left ventricle, decreased left ventricular ejection fraction, diffuse myocardial dyskinesia, and pulmonary hypertension were observed (29). Since that initial report, additional cases of COVID-19–related myocarditis have been diagnosed using cardiac MRI (CMR) (Tables 1 and 2) (2, 28, 30–36) and in postmortem analysis of fatal cases and endomyocardial biopsies (Tables 3 and 4) (37–40). In a study of 41 laboratory-confirmed COVID-19 patients who were admitted to a designated hospital in Wuhan, China, 12% of the patients demonstrated acute cardiac injury, which was defined as either an increase in cardiac biomarkers or the presence of new abnormalities on electrocardiography or echocardiography (41). An autopsy study of COVID-19 patients revealed mononuclear infiltrate, predominantly composed of lymphocytes, that was associated with focal myocyte necrosis (39). Additionally, a fatal case of fulminant myocarditis, which was confirmed by biopsy, was reported in a 2-year-old SARS-CoV-2–infected patient (42).

In the majority of patients, myocarditis presents concurrently with SARS-CoV-2–related respiratory symptoms. Nevertheless, delayed presentation of cardiac complications occurring weeks after initial symptomatic COVID-19 can also occur (43–45). Myocarditis documented by CMR may present as a postacute sequela of SARS-CoV-2 infection in up to 19% of individuals (46), and isolated myocarditis without concomitant respiratory disease has been reported as an atypical presentation of COVID-19 (47, 48). The subclinical presentation of ongoing or resolving myocarditis is also reported (49–51), and asymptomatic or mild disease with CMR findings suggestive of cardiac injury have been demonstrated among young competitive athletes with COVID-19 (51).

Myocarditis is now defined as a risk factor for increased mortality in patients with COVID-19 (3). Although the recognition that COVID-19 may cause acute myocarditis may facilitate early diagnosis and possible prevention of myocarditis-related mortality, lack of an understanding of the mechanism(s) by which SARS-CoV-2 contributes to myocarditis and cardiac damage hinders thorough management of this condition. Direct viral damage of cardiomyocytes, a hyperinflammatory state, and cytokine storm, which usually occur in severe cases, have been suggested as the main drivers of acute myocardial injury and myocarditis (Figure 1).

#### Direct viremic damage.

Among coronavirus family members, SARS-CoV and MERS-CoV are less likely to directly infect the myocardium. In a postmortem analysis of patients who died from SARS-CoV, the virus was not detected in the heart by immunohistochemistry or in situ hybridization (52). Another study that analyzed SARS-CoV RNA in postmortem tissue samples from 7 patients also suggested that SARS-CoV has less tropism for the heart than other coronaviruses (53). Additionally, in a 33-year-old patient who died from MERS-CoV, histopathological analysis of viral particles in different tissues revealed no remarkable viral particles in heart tissue (54). However, viral RNA was detected in heart samples collected from a transgenic mouse model expressing the MERS-CoV receptor, human dipeptidyl peptidase 4 (DPP4, also known as CD26) (55).

It remains controversial as to whether SARS-CoV-2 directly infects myocardial cells. Evaluation of SARS-CoV-2 RNA by quantitative reverse transcriptase PCR (RT-PCR) in 39 autopsy cases indicated viral presence in the myocardium of 24 cases, although this was not associated with infiltration of mononuclear cells in the myocardium, as would be seen in myocarditis (56). Another study reported lymphocytic myocarditis and viral RNA detection by RT-PCR in 1 out of 23 postmortem fatal COVID-19 cases (57). Electron microscopy of endomyocardial biopsy samples of a patient with COVID-19–related cardiogenic shock also revealed the presence of the virus in the myocardial interstitium (58). Finally, it has been shown that SARS-CoV-2 can directly infect human pluripotent stem cell–derived cardiomyocytes, which is contingent on ACE2 expression (59). In contrast to these findings, postmortem analysis of three patients who died from COVID-19 in China revealed that despite the presence of pathological changes, such as degeneration and necrosis of parenchymal cells in the heart, SARS-CoV-2 was not detectable by RT-PCR (60). Similarly, in two male patients, aged 36 and 39 years old, myocarditis was noted based on CMR, and endomyocardial biopsy demonstrated myocardial inflammation without necrosis or presence of viral genome by RT-PCR (61).

#### Cytokine storm and inflammatory response.

A number of reports suggest that the hyperinflammatory state that can occur in COVID-19 patients contributes to myocardial injury and increased mortality (Figure 1C) (62–64). Severe cases of COVID-19 commonly present with high plasma levels of inflammatory cytokines and a prolonged proinflammatory response, leading to extensive tissue damage (65, 66). Retrospective analysis of 191 COVID-19 patients in China revealed increased serum inflammatory markers, including TNF-α, C-reactive protein (CRP), ferritin, D-dimer, and IL-6, and cardiac damage markers, including troponin T (67). Comparison of laboratory tests between patients who died from COVID-19 and those who recovered suggested that CRP and procalcitonin levels were associated with mortality. Poor prognosis was linked to cytokine storm and a subsequent decrease in helper and suppressor T cells (68–70). Furthermore, a recent study that included whole-genome sequencing of 2244 patients with COVID-19 from 208 ICUs in the United Kingdom showed that critical and life-threatening COVID-19 has a genomic association with SNPs in mediators of organ inflammation genes, including those encoding tyrosine kinase 2, DPP9, and monocyte/macrophage chemotactic receptor CCR2 (71). These findings emphasize a critical role of hyperinflammation in COVID-19 pathogenesis. It remains to be elucidated whether such phenotypes contribute to increased risk of myocarditis or cardiovascular events.

A number of studies support a role for hyperinflammation in cardiac manifestations of COVID-19. Patchy mononuclear infiltration in epicardium, mainly CD4^+^ T cells, was observed in 15 out of 25 COVID-19 patients in a postmortem analysis of their hearts (72). Blood levels of troponin T, which correlates with higher mortality, showed a positive linear correlation with CRP, indicating a possible role for inflammatory response in COVID-19–related cardiac damage (73). Furthermore, a retrospective analysis of 353 COVID-19 patients, of whom 79 had myocardial injury, demonstrated that high neutrophil/lymphocyte ratio, D-dimer, lactate dehydrogenase, and inflammatory cytokines were positively associated with cardiac troponin I levels. Thus, these markers have the potential to predict patients at high risk of developing myocardial injury (74). In a case report of a 6-year-old boy, who presented with persistent fever and was later confirmed to have concurrent parvovirus B19 and SARS-CoV-2 infections, blood tests exhibited pancytopenia, hypertriglyceridemia, and hypocalcemia and elevated IL-6, D-dimer, CRP, procalcitonin, and cardiac biomarkers. Echocardiography showed decreased left ventricle systolic function, and CMR obtained 20 days after symptom onset revealed the presence of edema with no evidence of cardiomyocyte necrosis. The authors suggested a possible role of cytokine storm in myocardial injury rather than the direct injury by the virus (75). Finally, in April 2020, eight children from England with either a positive SARS-CoV-2 test or previous exposure to a COVID-19–infected family member presented with fever, shock, hyperinflammation, and myocardial involvement (76). Similar cases were subsequently reported in other countries (77, 78), and in May 2020, this condition, which has been proposed as a potential cause of myocardial injury, was termed multisystem inflammatory syndrome in children (MIS-C) by the CDC (79).

### Myocardial interstitial fibrosis

Diffuse and focal myocardial fibrosis in the hearts of patients with COVID-19 has been reported and can occur in the absence of cardiac symptoms. A study showed that 7 of 26 patients who recovered from COVID-19 but later developed cardiac symptoms displayed edema and fibrosis by late gadolinium enhancement in CMR (80). Diffuse interstitial fibrosis was noted on CMR in a case report of a 45-year-old female without a history of myocarditis who presented with palpitation and atypical chest pain 3 months after contracting COVID-19 (5). Similar findings of diffuse fibrosis were also reported in a previously healthy 49-year-old male who presented with dyspnea 6 weeks after the initial onset of COVID-19 symptoms (44). In addition, the autopsy result of 14 COVID-19 patients revealed focal myocardial fibrosis in 6 cases; however, all had a past history of myocardial infarction (MI) (81). A postmortem analysis of another 4 patients who died because of SARS-CoV-2 showed mild focal fibrosis in cardiac tissue in 2 of the patients (82). It is of note that one of these patients had a medical history of chronic lymphocytic leukemia and the other patient underwent renal transplantation 3 months before contracting the virus. In another study, analysis of cardiac tissue by endomyocardial biopsy in a patient with cardiogenic shock revealed low-grade inflammation with focal interstitial fibrosis (58). In contrast to these findings, a case series of 4 pediatric patients who were admitted to the ICU because of MIS-C revealed edema but no evidence of necrosis or fibrosis via CMR (83). Edema without fibrosis by CMR was also reported in 2 patients with myocarditis due to COVID-19 (61). It is important to note that in all these cases, it is not clear whether fibrosis existed before COVID-19 contraction or developed subsequently because of the infection. Moreover, the absence of a history of underlying heart disease does not exclude the possibility of past fibrosis in heart tissue.

Replacement of necrotic cardiomyocytes (as a result of myocarditis, vasculitis, and microinfarctions) by fibroblasts appears to be the main mechanism of fibrosis in COVID-19 patients. In addition, cytokine storm and infiltration of the myocardium with immune cells, which can potentially initiate fibroblast to myofibroblast conversion and subsequent matrix remodeling, are among other possible mechanisms of fibrosis in these patients (84).

### EC dysfunction and vasculitis

ECs play a role in the regulation of immune response, inflammatory reactions, coagulation, and platelet function. As a result, these cells are key players in various pathologies associated with COVID-19 (85, 86). EC dysfunction and vasculitis, although currently considered as one of the main cardiovascular complications of COVID-19, are also believed to be among other mechanisms that may underlie COVID-19–induced myocarditis.

**Figure 2:**
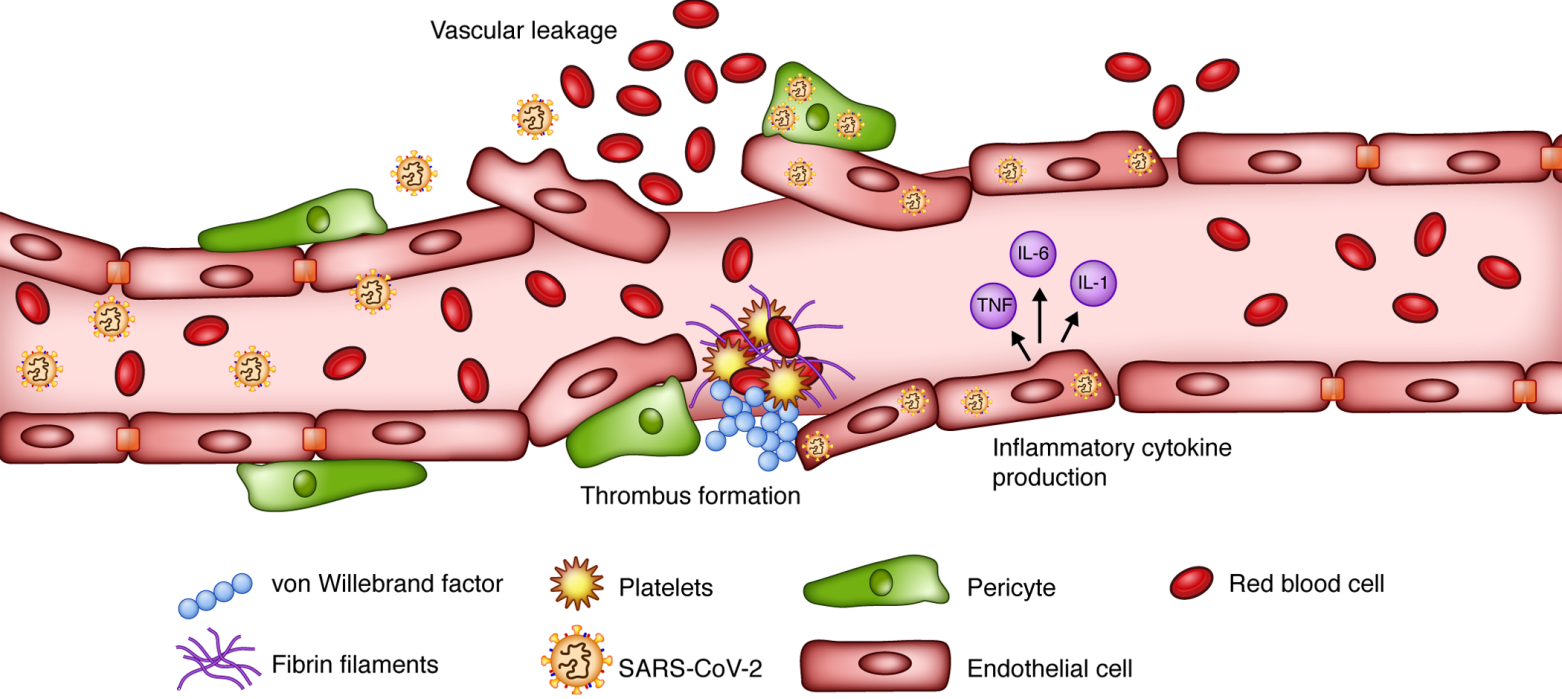
SARS-CoV-2 and endothelial cell dysfunction. Direct damage to endothelial cells (ECs) caused by SARS-CoV-2 disrupts cell integrity, resulting in EC activation and vascular leakage. Consequent exposure of vWF, which is involved in platelet aggregation and fibrin formation, leads to thrombus formation. Cytokines secreted by activated ECs can further augment the vascular inflammation, permeability, and leakage. Illustrated by Rachel Davidowitz.

A postmortem analysis of patients with COVID-19 demonstrated the presence of SARS-CoV-2 in the ECs of multiple organs (18) Direct viral infection of ECs, via SARS-CoV-2 receptors ACE2 and TMPRSS2 that are expressed on ECs (87), can lead to endothelial dysfunction and disruption of vascular integrity, causing subsequent leakage (Figure 2) (88). Hyperinflammation and hypercoagulability have also been reported as complications of EC dysfunction in COVID-19 patients (88). Postmortem studies demonstrated higher ACE2 expression in infected patients, which was associated with altered endothelial morphology, disruption of cell junctions, detachment of cells from the basement membrane, and cell swelling (89).

scRNA-Seq studies have demonstrated that genes associated with immunomodulation, leukocyte activation, cytokine production, and antigen presentation are expressed in ECs. Specifically, these transcripts are highly enriched in lung ECs compared with other organs (90). These results suggest that ECs may play a critical role in initiation and maintenance of inflammation. Furthermore, binding of SARS-CoV-2 to ACE2 impairs its enzymatic function, leading to bradykinin accumulation (87, 91), which is associated with increased vascular permeability (88). Thrombotic events are also commonly observed in hospitalized COVID-19 patients, especially those admitted to the ICU (92). Disruption of EC integrity exposes basement membrane to circulatory platelets, initiating platelet aggregation and thrombosis (88). Additionally, ECs express P-selectin, vWF, and fibrinogen in response to IL-1β and TNF-α, causing platelets to directly bind to ECs and become activated (93). Activated ECs are hypercontractile, which can lead to disruption of cell-cell junctions and vascular leakage.

Finally, a recent study showed that patients who contracted SARS-CoV-2 demonstrated EC dysfunction as shown by a 6% reduction in flow-mediated dilation (FMD) (94). A 6% reduction in FMD is clinically significant because every 1% reduction is associated with a 13% greater risk of a cardiovascular event (7).

### Thrombotic events

Thrombotic events are also commonly observed in hospitalized COVID-19 patients, especially those admitted to the ICU (92). Autopsy of 4 patients with COVID-19 revealed the presence of large emboli in the lungs and multiple microthrombi in other organs, including the brain (9). The presence of emboli has been associated with increased disease severity and mortality (67, 94–98). The underlying etiology of the prothrombotic state observed in patients with COVID-19 is multifactorial. As mentioned earlier, EC damage results in subsequent exposure of collagen within the extracellular matrix, leading to activation and recruitment of platelets (88). Additionally, activated ECs express a number of surface proteins, including P-selectin, that function as cell adhesion molecules to recruit platelets and leukocytes (93). An exaggerated inflammatory response, with elevated proinflammatory cytokines, also results in a predisposition to coagulopathy (9, 81, 99). Other factors that play significant roles in creating a hypercoagulable state and subsequent thrombus formation include disseminated intravascular coagulation, which commonly occurs in critically ill patients, and hyperferritinemia, which is associated with macrophage activation syndrome (100, 101). Additionally, ICU admission of patients requiring mechanical ventilation prolongs immobilization and venous stasis, which aggravates coagulopathy and frequently complicates disease course (96). Treatment with anticoagulants may lower mortality in hospitalized COVID-19 patients, as suggested by some studies (102).

Acute coronary events in COVID-19 patients have been reported since the emergence of the pandemic. In a systematic review of 1527 patients with COVID-19, 8% presented with acute MI, and MI risk was 13 times higher in patients with severe clinical symptoms (103). However, in a case series of 28 patients with COVID-19 who either presented with ST-elevation MI (STEMI) or developed STEMI during hospitalization, a lesion could not be identified in 40% of the patients who underwent coronary angiography (97). Similarly, out of 9 patients with COVID-19 who had STEMI, 3 patients displayed no obstructive disease (4). These observations suggest that these patients may have had acute thrombus formation that led to their MI. Other case reports also provide evidence that culprit lesions were not necessarily present in coronary arteries (19, 98); thus, this diagnostic challenge remains to be further elucidated.

### Cardiac arrhythmias

COVID-19 patients can also present with arrhythmia. Heart palpitations were reported as a presenting symptom in 7% of 137 individuals infected with SARS-CoV-2 hospitalized in Hubei province in China (104). In another report from China, the rate of arrhythmias in 138 confirmed COVID-19 cases was even higher at 16.7% (6). The most common arrhythmia observed in COVID-19 patients is sinus tachycardia. It is not clear whether sinus tachycardia is due to increased cardiac output secondary to fever, hypoxia, inflammatory stress, and medications or to myocardial structural changes (105).

A study of 700 patients admitted for COVID-19 infection reported 25 incidents of atrial fibrillation (AF), 9 bradyarrhythmias, and 10 nonsustained ventricular tachycardias (NSVTs). In addition, ICU admission was associated with incidents of AF and NSVT (106). Similarly, atrial arrhythmias were recorded on the ECGs of 27.5% of the patients admitted to the ICU compared with none of those who were treated in a non-ICU setting (107). Ventricular arrhythmias also occur in COVID-19 patients with critical conditions (108), making these patients vulnerable to cardiogenic shock, which requires further assessment to determine the need for extracorporeal membrane oxygenation (109). Medication side effects, inflammation of the myocardium, edema of the interstitial tissue, fibrosis, and myocarditis, leading to structural changes, conduction abnormalities, and dysregulation of ion channels (Na^+^ and K^+^), are among the underlying mechanisms by which cardiac arrhythmias happen in COVID-19 patients (110). Nonetheless, new occurrence of tachyarrhythmia accompanied by an elevation in serum cardiac biomarkers in a patient can be suggestive of myocarditis (63, 105).

### Dysautonomia

Dysautonomia is a medical condition caused by malfunction of the autonomic nervous system (ANS), generally due to the failure or overactivity of the sympathetic or parasympathetic components of the ANS. This condition has been reported in patients with COVID-19 and may occur as a severe acute manifestation of COVID-19 or as part of the chronic sequelae of extended disease referred to as “long COVID” (111–113). Recent studies suggest that some patients with long COVID may experience symptoms of autonomic dysfunction, especially postural orthostatic hypotension (POTS), which is defined by symptoms of orthostatic intolerance, including palpitation, headache, lightheadedness, fatigue, presyncope, shortness of breath, chest pain, sleep disturbances, and gastrointestinal symptoms upon upright position (114–116). Although the pathophysiology of POTS in COVID-19 remains to be elucidated, a number of mechanisms, including hypovolemia, invasion of the sympathetic nervous system and/or medullary centers in the brainstem, and autoimmunity, are among the potential underlying causes (114, 117).

### Medications

A wide range of compounds have been under investigation to treat COVID-19, but there currently is no specific treatment available for this rapidly spreading disease. A number of agents, including chloroquine and hydroxychloroquine, initially received emergency use authorization (EUA) from the FDA for treatment and/or prophylaxis of COVID-19. However, because of lack of clinical efficacy and side effects, especially cardiac adverse events, the FDA revoked EUA (118).

Among proposed treatment options, remdesivir is currently approved for use in hospitalized COVID-19 patients regardless of disease severity (119). Remdesivir is an antiviral agent with broad-spectrum activity against several viruses, including SARS-CoV and MERS-CoV. It is an adenosine analogue and a prodrug activated inside the cells via conversion to its pharmacologically active form, adenosine nucleoside triphosphate. The active form of remdesivir inhibits RNA-dependent RNA polymerase, thus resulting in RNA synthesis arrest (120, 121). Compassionate use of remdesivir has been tried in critically ill COVID-19 patients and has shown modest improvement in condition and shortened recovery times. However, because of insufficient information regarding its efficacy, trials are currently being conducted to assess the clinical impact of remdesivir (122). Remdesivir demonstrated cardiovascular side effects in 2 patients with COVID-19 who developed bradycardia, with QT interval prolongation and T wave abnormality in 1 patient (123). In addition, in a randomized controlled trial (RCT) conducted in China, 1 case of cardiac arrest was reported in a patient receiving remdesivir (120). Nevertheless, the data are insufficient to conclude whether or not remdesivir is safe, particularly in those with underlying cardiovascular disease. Thus, ongoing surveillance with an emphasis on cardiovascular aspects is needed in patients with COVID-19.

In contrast to the therapies with cardiovascular adverse effects that were initially intended to combat COVID-19, a number of agents have been studied for their favorable cardiovascular profiles in COVID-19 patients. Given the risk of thrombotic events in COVID-19 patients, use of anticoagulants has been recommended in all hospitalized patients with COVID-19, especially those with critical conditions who have no contraindication for anticoagulation (124). Colchicine is among other therapies that, because of its antiinflammatory properties, has been under investigation as a cardiovascular therapy in COVID-19 patients (125). A recently published meta-analysis of studies on colchicine demonstrated a significantly lower mortality rate with a possible lower risk of mechanical ventilation in patients with COVID-19 (126). The findings of the GRECCO-19 randomized clinical trial on the effect of colchicine on cardiac and inflammatory markers revealed a decrease in D-dimer and an improved clinical condition in patients who received colchicine (127). The results of ongoing randomized clinical trials will determine whether colchicine is effective in reducing cardiac injury in COVID-19.

In addition to aforementioned medications, ACE inhibitors, angiotensin receptor blockers (ARBs), and sodium glucose cotransporter-2 (SGLT-2) inhibitors are some of the drug classes that have been studied to be repurposed for COVID-19 treatment (128). The association of ACE inhibitors/ARBs with decreased mortality in cohorts of COVID-19 patients (129), along with their proposed mechanism in reducing viral entry in vitro (130), has prompted a number of RCTs on the effect of these therapeutics in COVID-19 patients (131). Nevertheless, there is not yet clear evidence regarding the clinical impact of ACE inhibitors/ARBs in COVID-19. In regard to SGLT-2 inhibitors, evidence suggests that they may have potential renoprotective and cardioprotective effects (132). To investigate the organ protection benefits of SGLT-2 inhibitors in COVID-19, an ongoing international, multicenter, randomized clinical study is evaluating dapagliflozin compared with placebo in prevention of COVID-19 complications or death and improvement of clinical recovery (Dapagliflozin in Respiratory Failure in Patients with COVID-19, DARE-19; https://clinicaltrials.gov NCT04350593).

## Conclusions

Since the emergence of COVID-19, multiple groups have reported cardiovascular complications associated with SARS-CoV-2 infection. A hyperinflammatory state and cytokine storm can lead to fulminant myocarditis. In addition, SARS-CoV-2 can potentially infect cardiomyocytes, ECs, and pericytes in the myocardium, leading to acute myocardial injury. Importantly, cardiac arrhythmias, the most common being tachyarrhythmias, and diffuse and focal fibrosis have been observed in COVID-19 patients. COVID-19 is also associated with a hypercoagulable state. Therefore, patients with COVID-19 should be monitored for cardiovascular events, especially patients with a past medical history of cardiovascular disease. Further investigations are required to screen and treat patients at risk of cardiovascular complications and to elucidate the mechanisms by which SARS-CoV-2 complicates the cardiovascular system.

Despite tremendous research on the cardiovascular complications of COVID-19 and its mechanisms, a number of unanswered questions remain to be addressed. Although high expression of ACE2 in endothelial cells and SNPs in certain inflammatory loci are associated with more severe disease and higher incidence of cardiac complications, a causal effect is yet to be determined. In addition, short- and long-term effects of COVID-19 in competitive athletes in whom the resumption of physical activity is important should be studied, and screening protocols to identify patients at risk of myocarditis should be developed. Because postacute sequela of SARS-CoV-2 infection is one of the presentations of COVID-19–associated myocarditis, future studies should also determine the target group, timing of the screening after initial recovery from COVID-19, and the tests that would help in distinguishing individuals at risk. Finally, given that COVID-19–associated cardiovascular complications can potentially be debilitating and sometimes life-threatening, routine screening protocols (including ECG, cardiac markers, echocardiography, or CMR and timing of each test based on each patient’s past medical history and predisposing factors) should be developed to identify patients at risk for myocarditis.

## Figures and Tables

**Table 4:**
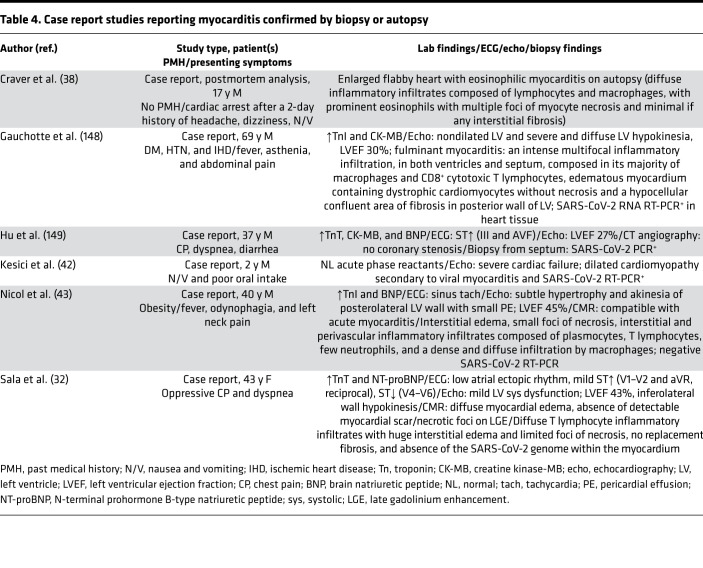
Case report studies reporting myocarditis confirmed by biopsy or autopsy

**Table 3:**
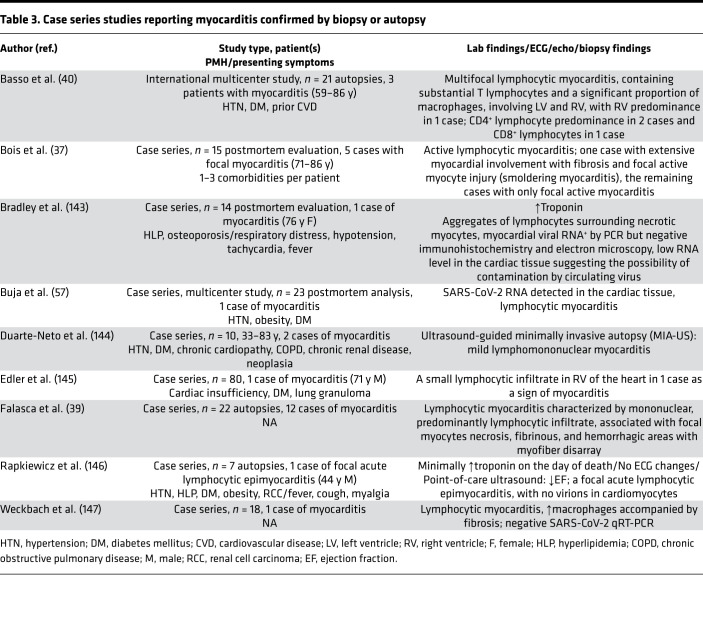
Case series studies reporting myocarditis confirmed by biopsy or autopsy

**Table 2:**
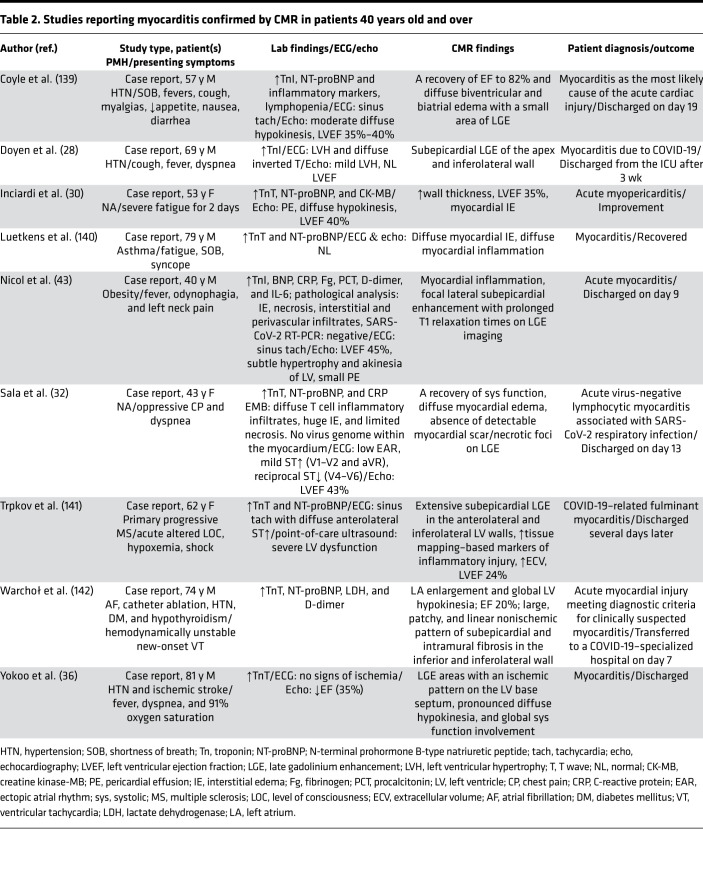
Studies reporting myocarditis confirmed by CMR in patients 40 years old and over

**Table 1:**
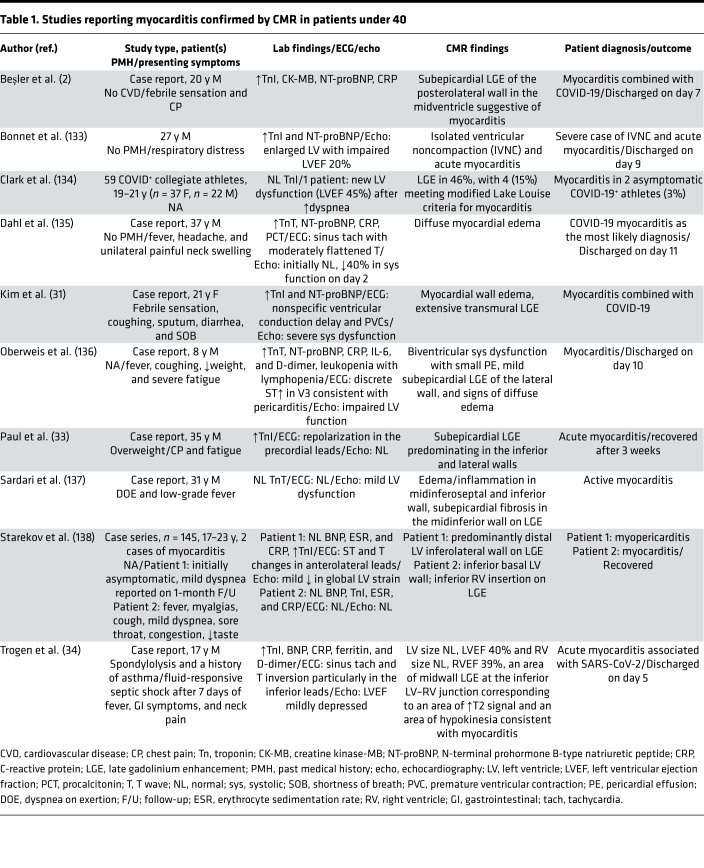
Studies reporting myocarditis confirmed by CMR in patients under 40

## Version 1 06/01/2021

In-Press Preview

## Version 2 07/08/2021

Electronic publication
